# Eag1 Gene and Protein Expression in Human Retinoblastoma Tumors and Its Regulation by pRb in HeLa Cells

**DOI:** 10.3390/genes11020119

**Published:** 2020-01-21

**Authors:** María de Guadalupe Chávez-López, Violeta Zúñiga-García, Blanca Elena Castro-Magdonel, Eunice Vera, Efraín Garrido, Janet Sánchez-Ramos, M. Verónica Ponce-Castañeda, M. de Lourdes Cabrera-Muñoz, Yesenia Escobar, Cindy Sharon Ortiz, Elisabeth Hernández-Gallegos, Arturo Avalos-Fuentes, Javier Camacho

**Affiliations:** 1 Department of Pharmacology, Centro de Investigación y de Estudios Avanzados del I.P.N., Mexico City 07360, Mexico; gchavez@cinvestav.mx (M.d.G.C.-L.); vzuniga@cinvestav.mx (V.Z.-G.); blanqfb@yahoo.com.mx (B.E.C.-M.); evera@cinvestav.mx (E.V.); lishergallegos@yahoo.com (E.H.-G.); 2 Hospital de Pediatría, Unidad de Investigación Médica en Enfermedades Infecciosas, CMN SXXI, IMSS, Mexico City 06720, Mexico; vponce@ifc.unam.mx; 3 Department of Genetics and Molecular Biology, Centro de Investigación y de Estudios Avanzados del I.P.N., Mexico City 07360, Mexico; egarrido@cinvestav.mx (E.G.); janetbiologia@gmail.com (J.S.-R.); 4 Department of Pathology, Hospital Infantil de México Federico Gómez, Secretaría de Salud, Mexico City 06720, Mexico; dra.cabreramalu@gmail.com; 5 Centro de Investigación Clínica Acelerada SC, Mexico City 07020, Mexico; yescobar@cicasite.com; 6 Departamento de Radioterapia, Hospital Regional de Alta Especialidad del Bajío, Gto., León 37660, Mexico; drasharonortiz@gmail.com; 7 Department of Physiology, Biophysics and Neuroscience, Centro de Investigación y de Estudios Avanzados del I.P.N., Mexico City 07360, Mexico; javalos@cinvestav.mx

**Keywords:** Eag1 channels, retinoblastoma, astemizole, tumor suppressor, potassium channels, Kv10.1, KCNH1

## Abstract

Retinoblastoma is the most common pediatric intraocular malignant tumor. Unfortunately, low cure rates and low life expectancy are observed in low-income countries. Thus, alternative therapies are needed for patients who do not respond to current treatments or those with advanced cases of the disease. *Ether à-go-go-1* (Eag1) is a voltage-gated potassium channel involved in cancer. *Eag1* expression is upregulated by the human papilloma virus (HPV) oncogene E7, suggesting that retinoblastoma protein (pRb) may regulate *Eag1*. Astemizole is an antihistamine that is suggested to be repurposed for cancer treatment; it targets proteins implicated in cancer, including histamine receptors, ATP binding cassette transporters, and Eag channels. Here, we investigated Eag1 regulation using pRb and Eag1 expression in human retinoblastoma. The effect of astemizole on the cell proliferation of primary human retinoblastoma cultures was also studied. HeLa cervical cancer cells (HPV-positive and expressing Eag1) were transfected with *RB1*. *Eag1* mRNA expression was studied using qPCR, and protein expression was assessed using western blotting and immunochemistry. Cell proliferation was evaluated with an MTT (3-(4,5-dimethylthiazol-2-yl)-2,5-diphenyltetrazolium bromide) assay. *RB1* transfection down-regulated Eag1 mRNA and protein expression. The human retinoblastoma samples displayed heterogeneous Eag1 mRNA and protein expression. Astemizole decreased cell proliferation in primary retinoblastoma cultures. Our results suggest that Eag1 mRNA and protein expression was regulated by pRb in vitro, and that human retinoblastoma tissues had heterogeneous Eag1 mRNA and protein expression. Furthermore, our results propose that the multitarget drug astemizole may have clinical relevance in patients with retinoblastoma, for instance, in those who do not respond to current treatments.

## 1. Introduction

Retinoblastoma is the principal pediatric intraocular malignant tumor [1]. Unfortunately, enucleation of either one or both eyes is commonly performed. Despite this, the overall survival in developed countries is very high, but low cure rates and low life expectancy are observed in low-income countries [2]. Therefore, alternative therapeutic approaches to treat these patients in low-income countries are needed. Patients who had a bilateral retinoblastoma in childhood have an elevated risk to develop other tumors [3,4]. However, why many other tissues seem not to be affected by the *RB1* mutations remains elusive. The retinoblastoma gene (*RB1*) was the first tumor suppressor to be described [5]. The corresponding encoded Rb protein (pRb) binds to several proteins including different transcription factors like E2F, snRNA-activating protein complex (SNAPc), TATA-binding protein (TBP), and Brahma-related gene (BRG) [6,7]. In addition, pRb regulates many epigenetic processes and has been suggested to be required to maintain chromosomal stability [8]. Accordingly, pRb has a very important role in cell proliferation, differentiation, and apoptosis, both in normal cells and different cancers [9]. Interestingly, retinoblastoma samples share a common miRNA expression profile, and some mRNA targets of these miRNAs include *RB1*, as well as other tumor suppressor genes [10].

*Ether à-go-go-1* (*Eag1*, *KCNH1*, *Kv10.1*) is a voltage-gated potassium channel displaying oncogenic properties [11]. Eag1 has a very restricted distribution in healthy tissues, and it is mainly expressed in the brain; slightly in the placenta, testes, adrenal glands; and transiently in myoblasts [12,13]. Eag channels have also been detected in the normal retina [13,14]. In contrast, *Eag1* is overexpressed in many cancer cell lines and human tumors, including those from the liver, cervix, lung, breast, and colon [11,13,15,16,17,18,19]. Interestingly, the human Eag-related channel (*Herg1*), which is very relevant for cardiac repolarization [20], has been found to be overexpressed in retinoblastoma samples from patients that did not benefit from conservative treatment, namely chemotherapy followed by cryotherapy and/or laser treatment [21]. *Eag1* has also been proposed as a novel anti-cancer target because the inhibition of either its gene expression or channel activity decreases the tumor cell proliferation in vitro and in vivo [22]. The second-generation anti-histamine astemizole is a very interesting molecule to be repositioned for cancer therapy because it targets different proteins involved in cancer, including histamine receptors and ATP binding cassette (ABC) transporters, as well as Eag1 and Herg channels [22,23,24,25].

*Eag1* mRNA expression was detected by our group in cervical biopsies from patients with human papilloma virus (HPV) infections [16]. Later, we also discovered that normal human keratinocytes do not express *Eag1* mRNA, but a very high *Eag1* expression was found when these cells were transfected with the HPV oncogenes E6 and/or E7 [26]. These observations led us to suggest that *Eag1* expression may be regulated by the p53 and Rb pathways, especially because the E7 HPV oncoprotein targets and inactivates pRb [9]. Actually, it has been reported that the p53-miR-34 pathway and E2F (one of the transcription factors targeted by pRb) regulate *Eag1* expression [27,28]. Herein, we investigated the regulation of Eag1 by *RB1* in HeLa cervical cancer cells because these cells are HPV-positive, express both *Eag1* channels and *RB1* [11,26], and are very suitable for transfection. In addition, we studied Eag1 expression in human retinoblastoma samples, as well as the effect of astemizole on the cell proliferation of human retinoblastoma primary cultures.

## 2. Materials and Methods

### 2.1. Cell Line and Reagents

The HeLa human cervical cancer cell line was obtained from the American Type Culture Collection (Manassas, VA, USA) and cultured in Dulbecco’s modified Eagle’s medium (DMEM) (Invitrogen, Carlsbad, CA, USA), supplemented with 10% FBS, and incubated at 37 °C in a 5% CO_2_ atmosphere. Astemizole was kindly provided by Liomont Laboratories (Mexico City, Mexico). Imipramine and DMSO were purchased from Sigma Chemical Co. (St. Louis, MO, USA). The anti-Eag1 antibody was purchased from Novus Biologicals, (Centennial, CO, USA), the anti-Rb antibody was purchased from Santa Cruz Biotechnology (Dallas, TX, USA), and the anti-actin antibody was purchased from Sigma Chemical Co. (St. Louis, MO, USA).

### 2.2. Cell Transfection

HeLa cells were transfected using Lipofectamine 2000 (Invitrogen, Carlsbad, CA, USA) following the manufacturer’s protocol. Briefly, 2 μg of the pCMV-*RB1* plasmid (a generous gift of Dr. Roberto Weinmann) in 100 µL Opti-MEM medium (Invitrogen, Carlsbad, CA, USA) were mixed with 5 µL Lipofectamine 2000 and incubated for 45 min at room temperature. The transfection mixture was then combined with 2 mL fresh medium (37 °C) and added to the cells. After an incubation period of 5 h, the transfection mixture was removed, cells were washed twice with PBS, and fresh medium was added; the incubation continued for 48 h. Control experiments were performed under the same conditions, but the cells were transfected with the empty vector (pcDNA3).

### 2.3. Human Tumor Samples and Primary Cultures

Retinoblastoma samples were obtained from 40 enucleated eyes from patients attending the Hospital de Pediatría CMN SXXI, Instituto Mexicano del Seguro Social (IMSS), and the Hospital Infantil de México “Federico Gómez”, and participating in a larger IRB approved case-series study [29]. The age of the children was 19.75 ± 14.7 months (range 2 to 60 months); twenty-four samples were obtained from girls and sixteen tissues from boys. All patient parents gave their informed consent for inclusion before the patients participated in the study. The study was conducted in accordance with the Declaration of Helsinki, and the protocol was approved by the Ethics Committee of the Hospital Infantil de México “Federico Gómez” (Children’s Hospital of Mexico “Federico Gómez”) (project identification code: HIM/2012/54 SSA 1042), and the Comisión Nacional de Investigación Científica (National Commission for Scientific Research), IMSS (project identification code: IMSS R2012-785-039). The children had not received any anticancer treatment. Tumors were collected and cultured in sterile conditions for a week in FBS 10% RPMI (Gibco^®^, Thermo Fisher Scientific, Waltham, MA, USA) medium. Thirty retinoblastoma primary cultures (15 unilateral and 15 bilateral) were used to obtain RNA. Samples intended for immunohistochemistry were fixed in paraformaldehyde. Ten retinoblastoma primary cultures (five unilateral and five bilateral) were used for cell proliferation assays. The primary cultures were used only once with no passages. A human pediatric normal brain obtained postmortem was used as a “control” for RNA expression studies.

### 2.4. Real-Time PCR

Total RNA was extracted with TRIzol reagent. Two micrograms of total RNA was reverse-transcribed using Moloney Murine Leukemia Virus Reverse Transcriptase (M-MuLV, M0253S, BioLab®, Inc., Lawrenceville, GA, USA). Real-time PCR was performed with 1 µL of cDNA using the TaqMan™ detection system (Applied Biosystem, Foster City, CA, USA) and the Universal PCR Master Mix reagents kit (Thermo Fisher Scientific, Waltham, MA, USA). *KCNH1* TaqMan probes for *Eag1* (ID: Hs00924320_m1) and *h-HPRT* (hypoxanthine-guanine phosphoribosyl transferase, Part Number 4326321E, constitutive gene) were used. The PCR protocol was 95 °C for 15 s and 60 °C for 1 min (40 cycles). The data were analyzed using the 2^−∆∆Ct^ method.

### 2.5. Cell Proliferation

Cell proliferation was assayed using the colorimetric method with a 3-(4,5-dimethylthiazol-2-yl)-2,5-diphenyltetrazolium bromide (MTT) cell proliferation kit I (Boehringer Mannheim GmbH), as previously described [26]. Briefly, the cells were incubated for 96 h in culture medium either alone or in the presence of astemizole (2 μmol/L, 5 μmol/L, or 10 μmol/L) (kindly provided by Liomont Laboratories, Mexico City, Mexico), imipramine (10 μmol/L), or DMSO as a vehicle (reagents were purchased from Sigma Chemical Co., St. Louis, MO, USA). Absorbance data were obtained with a microplate photometer (Sunrise Touchscreen, Tecan, Männedorf, Switzerland).

### 2.6. Immunochemistry

HeLa cells and retinoblastoma tissues were seeded on charged glass slides. Retinoblastoma and brain paraffin-embedded tissue sections on a charged slide were boiled for antigen retrieval and processed as previously described [26,30]. The anti-Eag1 antibody was used at a 1:500 dilution overnight at 4 °C. The slides were then incubated with a secondary biotin antibody (Bio SB, Santa Barbara, CA, USA) for 15 min, and the specific staining reaction was completed by incubating the slides in the presence of 3,3′-diaminobenzidine in a buffer reaction solution (Bio SB, Santa Barbara, CA, USA) and observed as brown immunostaining. Tissue sections were counterstained with hematoxylin (Agilent Dako, Santa Clara, CA, USA). The slides were observed using an Olympus IX51 microscope (Olympus Corporation of the Americas, Center Valley, PA, USA), and photographs were taken with an Olympus DP70 camera (Olympus Corporation of the Americas, Center Valley, PA, USA).

### 2.7. Western Blotting

Cells were washed, scraped, and centrifuged, and the obtained pellets were resuspended in a lysis buffer (20 mM MOPS(3-(N-morpholino)propanesulfonic acid)-NaOH pH 7.0, 150 mM NaCl, 1% sodium deoxycholate, 1% Nonidet P-40, 1 mM EDTA, 0.1% SDS) supplemented with protease inhibitors. Lysis was completed with the freezing–thawing process; the lysate was then centrifuged, and the supernatant was collected. Forty micrograms of protein was separated using SDS-PAGE (10%), transferred to a nitrocellulose membrane, and incubated with the corresponding antibody (anti-Eag1 antibody 1:750 (Novus Biologicals, Littleton, CO, USA), anti-Rb antibody 1:2000 ( Santa Cruz Biotechnology, Dallas, Texas, USA), and anti-actin antibody 1:100,000 (Sigma, Chemical Co., St. Louis, MO, USA). The relative protein quantification was performed with ImageJ software (National Institutes of Health, MD, USA [31].

### 2.8. Statistical Analysis

Analysis of variance followed by the Tukey–Kramer test were used to compare data between different experimental groups using GraphPad Prism software version 5.0 (San Diego, CA, USA). Data analysis from PCR experiments in human tissues was performed with the nonparametric Mann–Whitney test. Comparisons between two or more clinical parameters versus the control were determined with nonparametric ANOVA (Kruskal–Wallis test) and Dunn’s test for post hoc analysis. The analysis of the western blot data was performed with Student’s *t*-test. The *p*-values < 0.05 were considered statistically significant in all cases.

## 3. Results

### 3.1. RB1 Transfection Decreased Eag1 Expression in HeLa Cells

**Figure 1:**
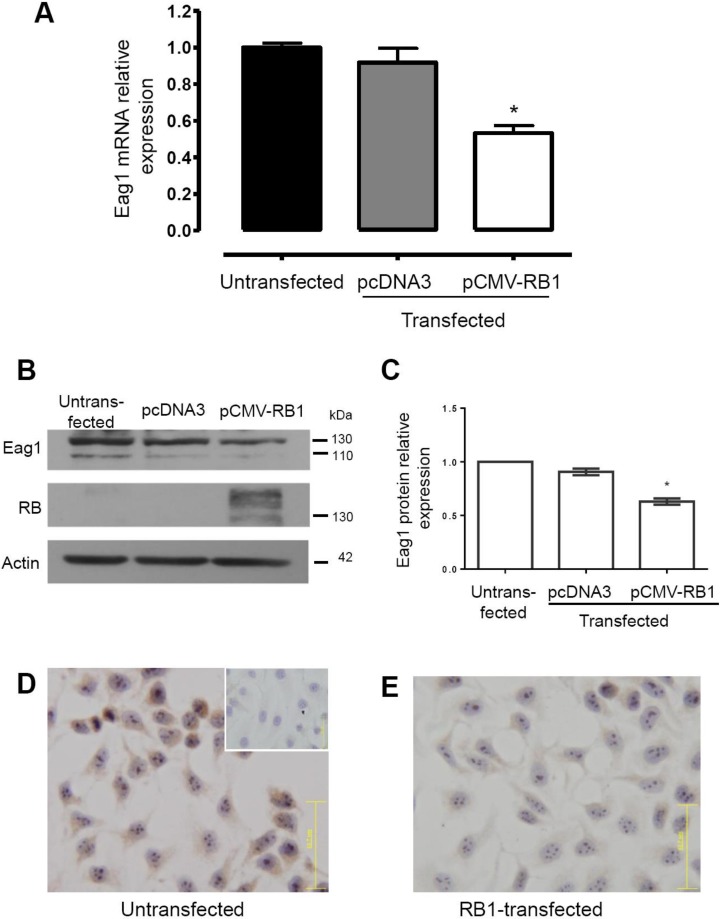
Retinoblastoma protein (pRb) down-regulates Ether à-go-go-1 (Eag1) expression in HeLa cells. Relative *Eag1* mRNA expression assessed using real-time PCR revealed a significant decrease of *Eag1* mRNA expression in cells transfected with *RB1*. The empty vector (pcDNA3) did not modify *Eag1* expression (**A**). Western blot experiments showed that pRb transfected cells display lower Eag1 protein levels and higher pRb expression (**B**). Quantitative analysis of both Eag1 bands, relative to actin expression (**C**). Accordingly, untransfected cells displayed clear Eag1 protein expression in immunochemistry assays (brown immunostaining) in the cytoplasm (**D**), while *RB1*-transfected cells showed decreased Eag1 protein expression (**E**). No signal was observed in the absence of the primary antibody (insert, (D)). The graphs show mean ± s.d., *n =* 3 in (A) and *n* = 5 in (C). * *p* < 0.05 versus untransfected and pcDNA3. Magnification: 400×.

First, we evaluated whether pRb regulated the expression of Eag1 by transfecting HeLa cells with *RB1*. Real-time PCR experiments showed that *RB1*-transfected cells exhibited a reduction in *Eag1* mRNA expression, while vector-only (pcDNA3)-transfection did not affect *Eag1* expression (Figure 1A). Western blot analysis demonstrated an increase in pRb and the concomitant decrease in Eag1 protein levels in the cells transfected with *RB1* (Figure 1B,C). Two Eag1 bands were observed, as expected, because of the reported glycosylated Eag1 form [32]. Immunocytochemistry assays confirmed that transfection with *RB1* decreased Eag1 protein levels (Figure 1D,E, brown immunostaining). Next, we investigated the Eag1 expression in human retinoblastoma samples.

### 3.2. Heterogeneous Eag1 mRNA and Protein Expression in Human Retinoblastoma Samples

**Figure 2:**
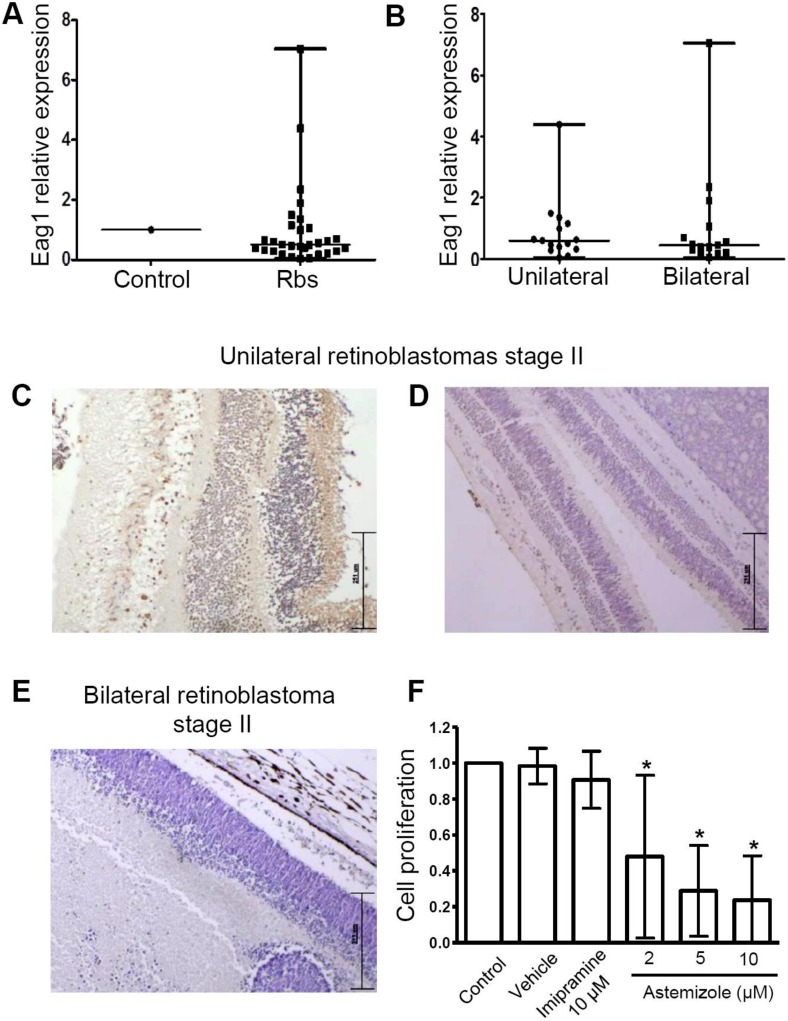
Eag1 was differentially expressed in human retinoblastoma and astemizole inhibited cell proliferation. (**A**) In comparison with the control (pediatric normal brain obtained postmortem, where the *Eag1* mRNA level was set to the value of 1, *Eag1* mRNA expression was very heterogeneous between the retinoblastoma samples. Many samples showed a lower expression, while other samples had clearly higher *Eag1* mRNA levels. No differences in *Eag1* expression versus the control was observed when separating the samples into unilateral (*n* = 15) and bilateral (*n* = 15) tumors (**B**). Examples of heterogeneous Eag1 protein expression studied using immunohistochemistry in three cases. Positive Eag1 expression in a zone of normal retina from an enucleated retinoblastoma case (**C**); weak Eag1 expression in retina and in tumor, upper right zone (**D**) and negative Eag1 expression (**E**). Magnification: 100×. Astemizole decreased the cell proliferation (assayed by metabolic activity) in retinoblastoma primary cultures (**F**) (*n* = 10). Each cell proliferation experiment was performed with sextuplicates. Panel (F) shows mean ± s.d. * *p* < 0.05 versus control.

To assess whether Eag1 was present in the retinoblastomas, *Eag1* mRNA expression was studied in 30 retinoblastoma samples using real-time PCR. Because of the difficulties in obtaining normal retinas from children, RNA from a human pediatric normal brain obtained postmortem from a child who died from non-oncologic causes was used as the “control.” The *Eag1* expression level found in the control tissue was set to a value of 1 to normalize the *Eag1* expression in the retinoblastoma samples. While the expression levels of the constitutive genes was very similar among samples, we observed that the *Eag1* mRNA level was very heterogeneous in the different samples. In comparison to the control, most of the retinoblastomas had a lower *Eag1* mRNA expression, but several samples displayed higher *Eag1* levels from 2–7 times that of the control (Figure 2A). Several samples showed *Eag1* mRNA levels that were very similar to that of the control. We also used commercially available mRNA from adult normal retina as a “control,” obtaining the same type of heterogeneous results, that is, *Eag1* mRNA levels were either below, similar, or higher than this control (data not shown). It is important to consider that the best comparison should be performed using normal retinas from children. The samples were then separated into unilateral and bilateral retinoblastoma; however, no statistically significant difference was found between tumor laterality and *Eag1* mRNA levels (Figure 2B). In accordance, Eag1 protein expression heterogeneity was also observed in retinoblastoma tissues (Figure 2C–E). When possible, Eag1 mRNA and protein expression were studied in the same sample, although in different tissue sections. *Eag1* mRNA levels did not always correlate with Eag1 protein expression. For instance, strong protein channel expression was found in one stage II unilateral retinoblastoma from a boy (Figure 2C, brown immunostaining, zone of normal retina shown), but this sample showed low *Eag1* mRNA levels. In another case, weak Eag1 protein expression was observed in the same type of tumor (unilateral retinoblastoma, stage II) but coming from a girl (Figure 2D); in this case, the very low protein expression correlated well with the low *Eag1* mRNA level in the same sample. No Eag1 expression was found in a girl with stage II bilateral retinoblastoma (Figure 2E); interestingly, this case exhibited high *Eag1* mRNA expression.

### 3.3. Astemizole Decreased the Cell Proliferation in Primary Retinoblastoma Cultures

Because many patients do not respond to chemotherapy, it is necessary to find novel therapeutic agents for these patients. Thus, we wondered whether two drugs that are widely used to inhibit Eag1, namely, astemizole and imipramine, might affect the cell proliferation of primary retinoblastoma cultures. The advantage of using these drugs is that they target several proteins involved in cancer, including potassium channels [22,23,24,25]. Ten primary retinoblastoma cultures were incubated with either astemizole (2, 5, or 10 μM) or imipramine (10 μM) for 96 h. While imipramine showed no effect, astemizole decreased the cell proliferation in a concentration-dependent manner (Figure 2F).

## 4. Discussion

New therapeutic approaches for patients with retinoblastoma who do not respond to current treatments or those with advanced disease, as well as molecular insights into the gene expression regulation by retinoblastoma, are needed. Here, we found that *RB1* transfection down-regulated the mRNA and protein expression of the oncogenic Eag1 potassium channel in HeLa cells. Experiments to study whether potassium currents and membrane potential are affected by *RB1* transfection are warranted. Because of the relevance of Eag1 in cancer, the pRb regulation of this channel may offer novel approaches that decrease Eag1 expression and subsequently inhibit cancer cell proliferation and tumor development.

We also observed that Eag1 had a heterogeneous distribution in retinoblastoma samples. To our knowledge, this is the first report of Eag1 expression in human retinoblastoma. We used mRNA from a child’s brain obtained postmortem to compare *Eag1* mRNA expression. Although the best control would be normal child retinas, they are very difficult to obtain. When possible, we studied Eag1 mRNA and protein expression from the same sample. We found a correlation between Eag1 mRNA and protein expression levels in only one out of three cases. Although it is common to find limited correlations between mRNA and protein levels in different systems [33], more studies are needed to determine, for instance, the half-life of Eag1 mRNA and protein in this type of tumor. Unfortunately, the small size of the tumors does not allow for several assays to be performed; however, more quantitative analysis of Eag1 protein expression (for instance, western blot experiments) in retinoblastoma samples is warranted. The different *Eag1* mRNA expression levels found between retinoblastoma samples did not correlate with tumor laterality or with other clinical features, such as the tumor stage or sex. This may be explained either by the intertumor heterogeneity or by the mutations in the *RB1* gene are diverse, producing truncated versions of pRb and differentially affecting its ability to associate with the many proteins it is able to bind [6,7,8]. More studies are needed to determine the potential association of Eag1 expression with prognosis, mutations in *RB1*, or response to chemotherapy in retinoblastoma. Moreover, the role of Eag1 in retinoblastoma should also be studied, for instance, in animal models. *Eag1* knockout mice have been generated [34]. These animals did not show any major abnormalities in the central nervous system level, and it would be very relevant to investigate how sensitive these mice are to develop retinoblastoma. Here, we investigated retinoblastoma tissues from children only; it would also be very important to study retinoblastoma tissues from adults.

We tested the effect of the multitarget Eag1 inhibitors astemizole and imipramine on the cell proliferation of primary retinoblastoma cultures. Although both drugs target Eag1, astemizole also targets other proteins involved in cancer, i.e., Herg1 channels, histamine receptors and ABC transporters [24]. Thus, the effect of astemizole on retinoblastoma cell proliferation may be due to its multitarget properties. More studies are needed to identify the precise molecular mechanisms and targets involved in the inhibition of cell proliferation by astemizole in retinoblastoma cells, as well as the effects of other astemizole concentrations and incubation times, to determine whether astemizole-resistant cells exist. It will also be very important to perform electrophysiological studies to characterize potassium channel activity in primary retinoblastoma cultures. Astemizole inhibits *Eag1* mRNA expression in breast and prostate cancer cells [35,36], therefore, it would be very interesting to study whether it has the same effect on primary retinoblastoma cultures. The investigation of the effect of other antihistamines on retinoblastoma cell proliferation is also warranted.

## 5. Conclusions

Our results suggest that Eag1 mRNA and protein expression is regulated by pRb in vitro. Heterogeneous Eag1 mRNA and protein expression is found in human retinoblastoma tissues; to our knowledge, this is the first report of Eag1 expression in human retinoblastoma samples. Our findings also suggest that the multitarget drug astemizole may have clinical relevance in patients with retinoblastoma, for instance, in those who do not respond to current treatments, or those with advanced cases of the disease.
